# 

**Published:** 1911-04-29

**Authors:** 


**The Medical Library.**

"Photomicrography." A Practical Guide and Introduction. 41 Reproductions of original specimens. 63 drawings illustrating instruments and methods. By Edmund Spitta, M.R.C.S., F.R.A.S. ... ... ... ... ... ... 12s. 0d.

"Clinical Diagnosis." A Practical Illustrated Handbook of Chemical and Microscopic Methods. By W. G. Aitchison Robertson, M.D., F.R.C.P. ... ... ... ... ... ... 3s. 9d.

"Notes on General Practice." Danger Signs and Common Pitfalls. By S. M. Hebblethwaite, M.D. ... ... ... ... ... ... 3s. 9d.

"Medical History from the Earliest Times." By E. Withington, M.A., M.B. Oxon ... ... ... 12s. 10d.

Of all booksellers or of The Scientific Press, Limited, 28 and 29 Southampton Street, Strand, London, W.C.

### BUILDINGS CONTEMPLATED AND IN PROGRESS.

Buxton.—Messrs. Bryden and Walton, of Buxton, are architects for the completion of a cottage hospital at Sherbrook. Tenders are invited.

London.—The Guardians of the Strand Union invite tenders for additions and alterations at the Receiving Workhouse and Casual Wards, Lincoln's Inn Fields. Mr. A. A. Kekwick, M.S.A., is architect and plans may be inspected at his office, 12 Norfolk Street, Strand. Tenders to be delivered not later than May 2.

Maidenhead.—It is recommended that a tender by Messrs. Lovell and Sons of £1,179 be accepted by the Town Council for the erection of new administration block, laundry, and disinfecting chamber.

Newark.—Tenders are being invited for an isolation hospital; plans and specifications may be seen at the surveyor's office, Town Hall Buildings.

Plymouth.—Messrs. Thornely, Rooke and Barrons are architects for the additions to the Workhouse Laundry for the Guardians. A tender of £1,007 by Mr. J. Paynter has been accepted. Nine tenders were sent in, the highest being £1,269.

Singapore, Straits Settlements.—The Singapore Municipal Commissioners have decided to erect an Isolation Hospital at an estimated cost of about £18,250. The camp will be divided in sections for cases of smallpox, cholera, and plague.

Southampton.—The management of the Royal South Hants and Southampton Hospital propose to erect a new out-patients' department. Tenders are invited, and are to be delivered before May 8.

Stourbridge.—Mr. H. E. Folkes is architect for a pavilion at the Stourbridge and Halesowen Isolation Hospital. The new pavilion will provide eighteen beds, and an application has been made for a loan of £2,125.

Wimbledon.—The Corporation invite tenders to be delivered before May 6 for the conversion of enteric block into a four-bed observation ward, the erection of boiler-house and shaft, and the erection of two Cornish boilers, with the necessary seatings, etc., at the Isolation Hospital, Gap Road.

---

Lord Grey, the Governor-General, has laid the corner-stone of the new General and University Hospital in Toronto, the estimated cost of which is $3,000,000 (£600,000).

The Metropolitan Asylums Board's Convalescent Hospital on the south side of the Thames has been changed from Grove Farm Hospital to Southern Convalescent Hopsital. The Board now has two convalescent hospitals, the Northern Convalescent Hospital (Winchmore Hill) and the Southern Convalescent Hospital (Dartford).

The Duchess of Hamilton and Brandon will lay the foundation stone of the new Nurses' Home for seventy-five nurses of the London Homœopathic Hospital, opposite the Hospital in Great Ormond Street, W.C., on Tuesday, May 23 next, at 2 o'clock. The Board of Management appeals for £8,000 to complete the building of the Home. Donations may be sent to the Secretary, Mr. Edward A. Attwood, at the Hospital, Great Ormond Street, W.C.

Colonel Douglas Wardrop has been appointed house governor and medical superintendent of King Edward's Convalescent Home for Officers at Osborne, in place of Lieut.-Colonel Kilkelly, who has resigned. This post is in the gift of the First Commissioner of Works and Public Buildings.

### APPOINTMENTS VACANT.

Full particulars of these vacancies can be obtained on reference to our advertisement columns:

Beckett Hospital, Barnsley.—Second House Surgeon.

Leeds General Infirmary.—Ophthalmic Officer.

Leeds General Infirmary (Ida and Robert Arthington Hospital.—Medical Officer, Obstetric Officer, Two House Physicians, One House Surgeon, One Ophthalmic House Surgeon.

Royal Albert Hospital, Devonport.—Assistant Resident Medical Officers.

Whitechapel Union Infirmary.—First Assistant Medical Resident Officer.

### COMING EVENTS OF THE WEEK.

[Communications for this column must be sent to 29 Southampton Street, Strand, before noon on Wednesday.]

Monday, May 1.—Costume Skating Fête at Maida Vale Rink on behalf of Prince Francis of Teck Memorial Fund, at 9 p.m.

Tuesday, May 2.—Polyclinic, Chenies Street: Dr. H. Tidy, Medical Clinique, at 4 p.m.

Dr. J. S. Fairbairn: "Displacements of the Uterus and Pelvic Floor," at 5.15 p.m.

National Hospital for Paralysed, Queen Square, W.C.: Clinical lecture, "Cerebral Diplegia," by Dr. James Collier, at 3.30 p.m.

Central London Throat Hospital, Gray's Inn Road: Lecture, "Pharynx and Naso-Pharynx," by Mr. I. G. French, at 3.45 p.m.

Wednesday, May 3.—League of Mercy: Meeting, Hitchin District, Mrs. Richardson and Miss Lucas "At Home," Old Town Hall, Hitchin, at 3 p.m.

Polyclinic, Chenies Street: Mr. E. Rock Carling, Surgical Clinique, at 4 p.m.; Dr. William Hill, "The Radium Treatment of Cancer in the Gullet and Stomach," at 5.15 p.m.

Thursday, May 4.—Polyclinic, Chenies Street: Dr. Cecil Wall, Medical Clinique, at 4 p.m.; Mr. Claud Worth, "Opthalmology in General Practice," at 5.15 p.m.

Festival Dinner of the Royal Hospital for Incurables, Putney Heath, at the Grocers' Hall, Princes Street, 6.30 for 7 p.m. The Hon. Harry Lawson in the chair.

Friday, May 5.—Central London Throat Hospital, Gray's Inn Road: Special Lecture, "Nasal Obstruction, its Causes, Consequences, and Cure," by Mr. Stuart-Low, at 3.30 p.m.

National Hospital for Paralysed, Queen's Square, W.C.: Clinical Lecture, "Subacute Combined Degeneration of the Spinal Cord," by James Collier, at 3.30 p.m.

Polyclinic, Chenies Street: Mr. Edward Clarke, Clinique (Eye), at 4 p.m.

